# Comparison of Smoothness, Movement Speed and Trajectory during Reaching Movements in Real and Virtual Spaces Using a Head-Mounted Display

**DOI:** 10.3390/life13081618

**Published:** 2023-07-25

**Authors:** Norio Kato, Tomoya Iuchi, Katsunobu Murabayashi, Toshiaki Tanaka

**Affiliations:** 1 Department of Physical Therapy, Faculty of Health Sciences, Hokkaido University of Science, Sapporo 006-8585, Japan; 2 Shin-Sapporo Orthopaedic Hospital, Sapporo 004-0051, Japan; 2194016@stu.hus.ac.jp; 3 Division of Rehabilitation Sciences, Graduate School of Health Sciences, Hokkaido University of Science, Sapporo 006-8585, Japan; 9233202@hus.ac.jp; 4 Sapporo Keijinkai Rehabilitation Hospital, Sapporo 060-0010, Japan; 5 The Research Center for Advanced Science and Technology, Institute of Gerontology, The University of Tokyo, Tokyo 113-8656, Japan; t.tanaka@iog.u-tokyo.ac.jp

**Keywords:** virtual reality, body coordinate system, movement smoothness

## Abstract

Virtual reality is used in rehabilitation and training simulators. However, whether movements in real and virtual spaces are similar is yet to be elucidated. The study aimed to examine the smoothness, trajectory, and velocity of participants’ movements during task performance in real and virtual space. Ten participants performed the same motor task in these two spaces, reaching for targets placed at six distinct positions. A head-mounted display (HMD) presented the virtual space, which simulated the real space environment. The smoothness of movements during the task was quantified and analysed using normalised jerk cost. Trajectories were analysed using the actual trajectory length normalised by the shortest distance to the target, and velocity was analysed using the time of peak velocity. The analysis results showed no significant differences in smoothness and peak velocity time between the two spaces. No significant differences were found in the placement of the six targets between the two spaces. Conversely, significant differences were observed in trajectory length ratio and peak velocity time, albeit with small effect sizes. This outcome can potentially be attributed to the fact that the virtual space was presented from a first-person perspective using an HMD capable of presenting stereoscopic images through binocular parallax. Participants were able to obtain physiological depth information and directly perceive the distance between the target and the effector, such as a hand or a controller, in virtual space, similar to real space. The results suggest that training in virtual space using HMDs with binocular disparity may be a useful tool, as it allows the simulation of a variety of different environments.

## 1. Introduction

Virtual Reality (VR), represented by Metaverse, has evolved into a popular and widely used technology, owing to its ease of use and simplified content creation, making it accessible to a broad audience. One of the key reasons for the growing popularity of VR is the availability of game engines such as Unity. These engines have made the development process effortless, enabling non-specialists to develop applications that would otherwise require specialized skills and knowledge [1]. Commercially available 360-degree cameras can help create VR images without requiring advanced computer graphics expertise, which in turn lowers barriers to VR content creation [2]. Moreover, the performance and cost of dedicated VR devices such as head-mounted displays (HMDs) and VR goggles that use smartphones have improved significantly, making the VR experience more accessible and affordable than ever before. These factors have contributed to the widespread adoption of VR, making it a promising technology with a bright future ahead [3].

VR is defined as “the use of interactive simulations created with computer hardware and software to present users with opportunities to engage in environments that appear and feel similar to real-world objects and events” [4]. VR has been used in a wide range of fields (including education, medicine, and entertainment) because of its ability to simulate a variety of environments [5,6,7]. In medicine, especially in rehabilitation, it has been effectively applied to the treatment of various diseases, such as Parkinson’s disease, stroke, orthopaedic diseases, and higher brain dysfunction [8,9,10,11,12,13]. The advantages of using VR for rehabilitation include cost-effectiveness [14], the possibility of adjusting variables important for rehabilitation such as difficulty and motivation [15,16], and real-time multisensory interactions [17]. In addition, virtual spaces have the advantage of presenting a variety of stimuli, including unusual images, and enable the quantitative evaluation of movements. Furthermore, remote rehabilitation using VR has also been conducted, and its effectiveness has been demonstrated [18,19].

However, a challenged posed by VR technology lies in the disparity between the sensory information received and the information derived from a real space. The differences in sensory information between the two spaces may influence human movement. For example, studies of postural control in virtual space have reported increased instability and alternative postural control strategies compared to real space [20,21,22]. One specific type of sensory information that differs between real and virtual spaces is depth perception. The cues used for depth perception can be classified into pictorial (such as perspective and skin texture gradient) and non-pictorial (such as convergence, adjustment, and binocular disparity) [23,24]. For pictorial cues, it is possible to approximate the information obtained from a real space by increasing the resolution of displays and HMDs and by using high-precision computer graphics. However, non-pictorial cues are affected by hardware limitations and thus may elicit different depth perceptions compared to those present in real spaces. For example, Renner et al. reported a tendency to underestimate distances in a VR space compared to a real space [25]. We examined the effect of different depth information using 2D and 3D monitors on the performance of reaching movements. The results showed that the 3D monitor with pseudo depth information could enable smoother reaching [26]. This result may be due to the fact that the visual information obtained from the 3D monitor includes physiological factors derived from the binocular disparity in addition to the psychological depth information obtained from the 2D monitor, such as perspective, skin texture gradient, and shading. When reaching motions in real and VR spaces were compared, we found that the performance was improved in real spaces [27]. The reason for this may be the difference in the coordinate system used to predict the distance to a given object. In a real space, it was predicted that subjects would perform the reaching operation by estimating the distance to the object in the coordinate system centred on themselves. However, when using a 3D monitor, the participant may have had to estimate the distance between the object and the coordinate system whose origin is the avatar projected on the display. Therefore, in terms of predicting distance, it is necessary to use a different method to that used in real space, making it more difficult to measure the distance to a given object.

HMDs provide a possible solution to the problem of mismatched viewpoint coordinate systems by estimating object distances using a self-centred coordinate system, similar to real space. This study aimed to investigate how the performance of reaching movements is affected when the viewpoints in real and virtual space are unified using an HMD.

## 2. Materials and Methods

**Figure 1:**
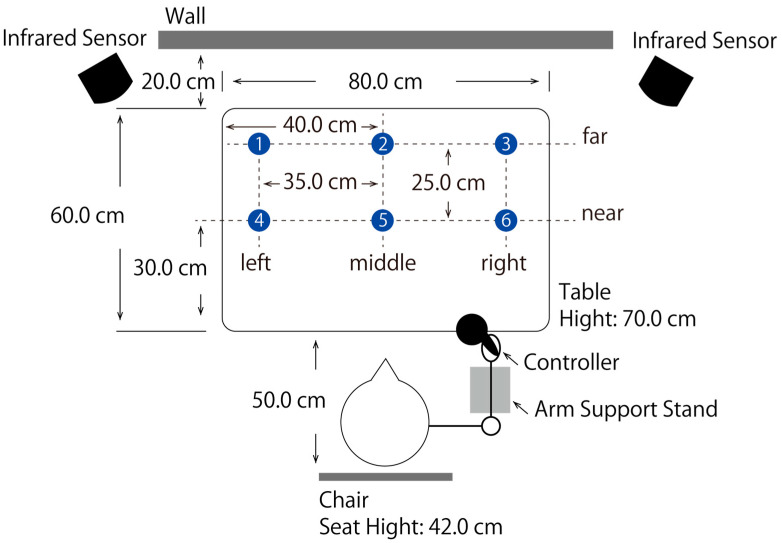
Experimental setup in real and virtual space conditions. The object to be reached was randomly placed at the six locations indicated on the diagram by numbers.

In this study, subjects were tasked with reaching a static object placed at the same distance and height in a real and in a virtual space. The details of the procedure are described below (Figure 1).

### 2.1. Participants

Ten healthy adults without orthopaedic or neurological disorders participated in this study (nine men and one woman; all subjects’ age: 21.0 years). The recruited individuals received no monetary compensation for their participation in the experiment. All the participants were right-handed. The dominant hand was defined as the upper limb used to throw a ball. All the participants had normal or corrected-to-normal vision. Patients with a history of musculoskeletal or central nervous system disorders that could affect the reaching movement were excluded. In addition, those who had difficulty wearing the HMD because of the use of glasses in their daily lives were excluded. All protocols for this study were approved by the Institutional Review Board of the Hokkaido University of Science (approval review No. 646). Informed consent was obtained from all the participants in accordance with the 1964 Declaration of Helsinki.

### 2.2. Equipment and Environment

**Figure 2:**
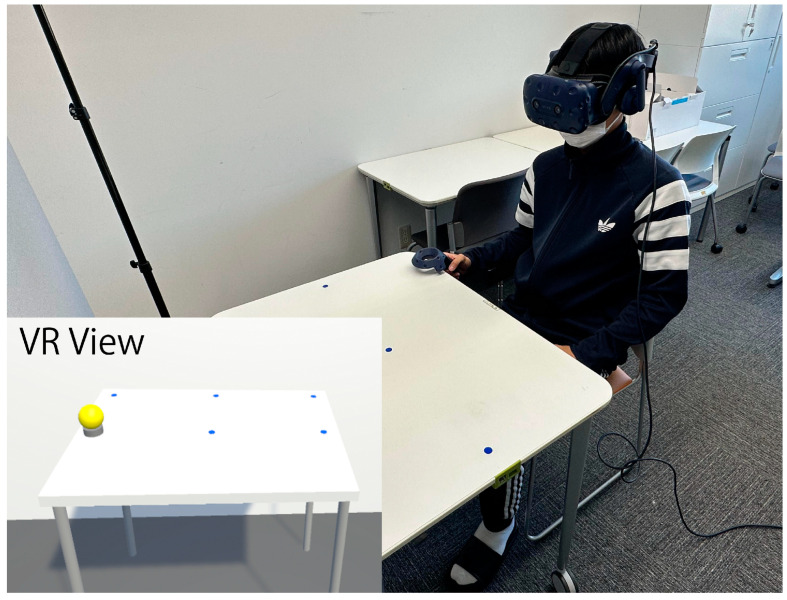
Image of the experimental scene in virtual space condition. The bottom left image is shown to the participant through the head-mounted display in the virtual space condition. The blue circles on top of the virtual table represent the target locations and the yellow ball represents the object to be reached.

Figure 1 shows the experimental environment. The distance from the table (WHD: 80.0 cm × 70.0 cm × 60.0 cm) placed in front of the wall to the backrest of the chair (seat height: 42.0 cm) was 50.0 cm. The forearm support was placed on the subject’s dominant hand side, and the height and placement points were adjusted so that the starting limb position of each subject was identical. Blue circle stickers (diameter: 2.0 cm) were placed on the desk at the six locations shown in Figure 1. For target placement in the depth direction, the row located at the centre of the table depth was defined as the near condition and the row 25.0 cm away from the near condition was defined as the far condition. For the horizontal target placement, the row located at the centre of the table width was defined as the middle condition, and rows 35.0 cm away from the middle condition to the left and right were defined as the left and right conditions, respectively. The six target placement locations were defined as far-left, far-middle, and far-right from the far-left position, and near left, near-middle, and near-right from the left side of the front. In real space, the target for reaching was a yellow ball (diameter: 7.0 cm) placed on a circular base (hight: 1.0 cm). For the measurement, the target was placed in such a way that the centre of the base coincided with the centre of the circular sticker. As shown in Figure 2, a virtual space similar to a real environment was created using the Unity software (Editor version 2020.3.26f1; Unity Technologies, San Francisco, CA, USA).

An HMD (VIVE Pro; HTC Inc., New Taipei City, Taiwan) was used to present the VR environment (Figure 2). The HMD had two 3.5-inch (diagonal) active-matrix organic light-emitting diodes (AMOLED) with a resolution of 1440 × 1600 pixels per eye and a refresh rate of 90 Hz. The field-of-view (FOV) of the HMD was 110° under optimal conditions. The attached controller had a built-in vibrator and provided vibration feedback when in contact with an object in the VE.

The spatial coordinates of the controller can be measured using the infrared sensors of the HMD system (BASE Station 2.0; HTC Inc., New Taipei City, Taiwan). In this study, the participants’ hand movements during reaching tasks in both real and virtual space were measured using the controller’s spatial coordinates. The controller coordinate data were stored in CSV format for subsequent data analysis.

In the virtual environment, the controller provided feedback to the participants by vibrating when it touched the target, simulating the sensation of touching a ball. For the collider setup in Unity, the target was represented by a sphere (diameter: 7.0 cm), and a sphere (diameter: 1.0 cm) was placed at the tip of the controller.

### 2.3. Procedure

As a common condition for both the real and the virtual environment, the participants performed reaching with their dominant hand holding the controller while seated in a chair. In the virtual space condition, the subjects wore the HMD. At the beginning of the experiment, the upper limbs were placed on the forearm support, with the dominant upper arm in an intermediate position of shoulder flexion/extension and mild abduction and the elbow in a 90-degree flexion position.

Prior to the experiment, the controller’s coordinates were measured for 5 s at the starting position to determine the stillness of each participant. In both environments, the participants were asked to start the task after the experimenter verbally signalled the start of the measurement. The participants were instructed to touch the yellow ball on the table using the tip of the controller, and to do so in a way that was comfortable for them without inhibiting their trunk movements. Additionally, they were asked to maintain their posture for 5 s after the controller made contact with the target.

The real and virtual space experiments were conducted on two separate days in random order for each participant. Measurements of the two space conditions were taken 2–3 days apart depending on the individual. Each experimental session consisted of participants reaching for targets positioned at six different locations. To facilitate task comprehension and the difference between the two environments, three practice trials were conducted, which relieved participants from performance concerns regarding their reaching. Subsequently, a series of five performance measurement trials were conducted, the first two for familiarization of the measurement procedures and the last three for data analyses.

### 2.4. Data and Statistical Analysis

This study investigated the differences between reaching movements in real and virtual space, focusing on the smoothness, trajectory, and speed of the reaching movement. The controller’s spatial coordinates during the reaching operation were used to calculate each parameter.

The smoothness of movement can be quantified using a concept known as jerk cost. Jerk is defined as the rate of change in acceleration over time. As the jerk cost decreases, the smoothness of the motion increases. In this study, the normalised jerk cost (NJC) was calculated using Equation (1) [28]:

(1) $$NJC=\sqrt{\frac{1}{2}\times \int_{t_{1}}^{t_{2}}{\left(\frac{d^{3}x}{dt^{3}}\right)}^{2}+{\left(\frac{d^{3}y}{dt^{3}}\right)}^{2}+{\left(\frac{d^{3}z}{dt^{3}}\right)}^{2}dt\times \frac{{\left(t_{2}-t_{1}\right)}^{5}}{{\left(length\right)}^{2}}}$$

t_1_: Start of Movement, t_2_: End of Movement
$\frac{d^{3}x}{dt^{3}},\frac{d^{3}x}{dt^{3}},\frac{d^{3}x}{dt^{3}}:$ the third derivatives of the spatial coordinates x, y and z
length: Length of Reach Trajectory
Length of Reach Trajectory $=\sum_{i=1}^{n-1}\sqrt{{∆x}_{i}^{2}+{∆y}_{i}^{2}+{∆z}_{i}^{2}}$
$∆x_{i}=x_{i-1}-x_{i}$, $∆y_{i}=y_{i-1}-y_{i}$, $∆z_{i}=z_{i-1}-z_{i}(i=1,2,3,\ldots ,n-1,n:Samplingnumber)$

The NJC was calculated using the controller coordinates from the starting position to the position where the target was touched. The start of the reaching motion was defined as the point at which the controller velocity exceeded the mean velocity + two standard deviations of the stationary state, and the end of the motion was defined as the point at which the controller velocity decreased below the mean velocity + two standard deviations.

To analyse the trajectory of the reach movement, the actual trajectory length was normalized by the shortest distance between the start and end of the movement. For the velocity of the reach movement, an analysis was performed to determine where the maximum velocity occurred during the reach movement, with 0% at the start and 100% at the end of the reach movement.

Statistical analysis was performed using SPSS software (version 28; IBM, Chicago, IL, USA). The normality of the data was assessed using the Shapiro–Wilk test. A paired t-test for parametric data and a Wilcoxon rank sum test for non-parametric data were used to detect differences between the real and virtual environments. Effect sizes were calculated using Cohen’s d for parametric data and Cliff’s delta for non-parametric data, using R software (version 4.2.1). The significance level was set at *p* = 0.05.

## 3. Results

The representative values for NJC, trajectory length ratio, and peak velocity time for reaching movements in both real and virtual space for all targets are shown in Table 1. Statistical analysis results showed no significant differences between the two spaces regarding NJC and time of peak velocity (NJC: df = 180, Z = −1.097, *p* = 0.273) (time of peak velocity: df = 180, t = −0.179, *p* = 0.858). However, a significant difference was observed for trajectory length ratios (df = 180, Z = −2.71, *p* = 0.007). The effect size of Cliff’s delta was 0.09, indicating a negligible effect size.

Table 2 shows the results of the statistical analysis for individual targets. No significant differences were found between the two spaces for NJC (df = 30; far-left target, Z = −1.183, *p* = 0.237; far-middle target, Z = −0.668, *p* = 0.504; far-right target, Z = −1.820, *p* = 0.069; near-left target, Z = 0.113, *p* = 0.910; near-middle target, Z = 0.195, *p* = 0.845; near-right target, Z = −1.203, *p* = 0.229). However, significant differences were observed in trajectory length ratios for the near-left and near-middle targets (df = 30: far-left target, Z = −0.072, *p* = 0. 943; far-middle target, Z = −0.237, *p* = 0.813; far-right target, Z = −1.656, *p* = 0.098; near-left target, Z = −2.347, *p* = 0.015; near-middle target, Z = −2.067, *p* = 0.039; near-right target, Z = −0.607, *p* = 0.544). The effect sizes of the Cliff delta for the near-left and near-middle targets were 0.015 and 0.039, respectively, indicating small effects. Furthermore, significant differences were observed in time of peak velocity for the far-left and near-right targets (df = 30: far-left target, Z = −0.072, *p* = 0. 943; far-middle target, Z = −0.237, *p* = 0.813; far-right target, Z = −1.656, *p* = 0.098; near-left target, Z = −2.347, *p* = 0.015; near-middle target, Z = −2.067, *p* = 0.039; near-right target, Z = −0.607, *p* = 0.544). The effect sizes of Cohen’s d for the far-left and near-right targets were 0.65 and 0.53, respectively, indicating a medium effect size.

## 4. Discussion

This study aimed to investigate the effects of differences between real and virtual space on reaching movements; specifically, the effect of presenting virtual space conditions using an HMD, which offered an equivalent viewpoint to that of real space. The results showed no significant differences in the smoothness of reaching movements and the time of peak velocity between the two spatial conditions. However, significant differences were observed for path length ratio, albeit with negligible effect sizes. Regarding the six target placements, there were no significant differences in the smoothness of the reaching movement. Conversely, significant differences in path length ratio and peak velocity time were observed at several locations, with small or medium effect sizes. These results suggest that the difference between the two spaces mildly to moderately effect movement speed and trajectory depending on the object’s placement, but less on the smoothness of the movement.

A possible explanation as to why there was no significant difference in the smoothness of the reaching motion between the two spaces is that physiological depth information was presented. The physiological factors associated with depth perception include accommodation, convergence, and binocular disparity. Of these three, accommodation and convergence are considered effective for depth perception, mainly for depth percept-ion up to 2.0 m from the observer [23]. However, the structure of the display device used in VR causes a discrepancy between the information provided by these two factors, which affects distance perception [29]. Extensive research has demonstrated a strong association between binocular disparity and depth perception [23,30,31]. In previous studies, we measured the performance of reaching movements towards objects in a virtual space, presented on both a 2D and a 3D monitor [26]. Three-dimensional monitors can present physiological depth information with binocular disparity, although it is pseudo. The results demonstrated enhanced smoothness in the reaching movement when the visual information was presented on the 3D monitor, suggesting that depth information by binocular disparity plays an important role in reaching movements. The HMD used in this study can adjust binocular disparity based on each participant’s interpupillary distance, thereby allowing for the presentation of depth information that more closely resembles reality. This adjustment may have been the reason why the smoothness of the reaching movement was not significantly different from that in real space. However, some studies have concluded that there is no difference in depth perception between stereopsis and flatness, or between binocular and monocular vision [32,33,34,35]. Therefore, further verification is warranted.

Another relevant factor that may have influenced the outcome was the use of an HMD, which enabled the participants to obtain visual information from the same perspective as if they were present in the virtual space. When the virtual space is presented from a first-person perspective, the HMD immerses the participants in a state where they are within the coordinate system of the virtual space. This allows the participants to directly perceive the distance between an effector, such as an avatar’s hand or a controller, and an object, using themselves as a reference point, similar to real space. In contrast, when a monitor or screen is used, participants have to indirectly estimate the distance based on the displayed images of the effector and the object. The participant must then translate this information to control their own movements in real space. Previous studies have shown that exercise in unusual environments requires the recalibration of visual and proprioceptive feedback, resulting in reduced motor performance [36]. For example, previous research has shown that display devices can cause temporal and spatial differences in behaviour in virtual space. Govindarajan et al. created a simulator to manipulate a wheelchair in a VR environment and examined the difference in performance between an HMD and a computer monitor [37]. The results showed that the HMD condition reduced the task completion time for wheelchair manoeuvring within a narrow space. Wenk et al. asked participants to perform a reaching task in a VR space with a computer monitor and two types of HMDs that can present VR and augmented reality environments and compared the performance and cognitive load associated with each display device [38]. They reported that the reaching movements were straighter, shorter in execution time, and smoother with less velocity change when HMDs were used instead of monitors. The results also indicated that the HMD reduced the cognitive load required of the participant, as the latency to start the movement was shorter. In contrast, Magdalon et al. conducted a comparative study that involved grasping movements in real and virtual spaces using an HMD and found differences between the two spaces [39]. One reason for this is the narrow FOV of the HMD. The FOV of the HMD used in this study was larger, which may have prevented differences between the two environments. However, some previous studies have shown that the shape of the object also changes the motion between spaces [39]; thus, further verification is needed.

Analysis of individual performances showed significant differences in three of the 10 participants recruited for this study. Two of these participants showed smoother movements in real space, while the other showed the opposite. According to interviews with the participants, in all cases this was their first experience of a virtual environment using an HMD. Conner, in his review of Virtual Reality Induced Symptoms and Effects (VRISE), noted the need for repeated exposure to VR to adapt to the sensory mismatches that occur in virtual space [40]. In this study, the number of training sessions within the virtual environment was set at three for each target coordinate, which may not have been sufficient to fully adapt to the environment. In the future, it will be necessary to determine the optimal number of training sessions per day for successful adaptation to the VR space. As some of the participants showed improved performance in the virtual space condition, it will be necessary to determine how often this occurs and what factors are common to individuals showing this improvement by increasing the total number of participants.

Significant differences were found in the trajectory and speed of the reaching movements for certain target coordinates. As participants were not given detailed instructions regarding the reaching movements in the two spaces, it is possible that the strategies used during the reaching movements differed in each condition. However, no significant differences were observed in movement smoothness, suggesting that acceleration and deceleration during reaching movements were performed appropriately within the virtual space conditions.

This study has four potential limitations. First, we were unable to recruit a sufficient number of participants because of the COVID-19 pandemic. As mentioned earlier, there were cases in which significant differences were found in the analysis by individual performance; therefore, we will continue to increase the sample size for further validation. The second limitation is the possibility that differences may have occurred in parameters other than those measured in this study—for example, the movement of the upper limbs and trunk during the reaching movement. In the future, we intend to increase the number of measured parameters and to evaluate potential differences between the two environments. The third limitation is the range of the reaching motion. In this study, target placement was limited to a distance range that allowed the participant to reach the target without having to move the trunk significantly, and to a certain height. In the future, it will be necessary to evaluate the effects of changing the range and height of the targets so that trunk and hip flexion are required. Fourth, this study is limited to reaching movements towards static objects. For moving objects, motion parallax information is important in addition to depth information, which may differ from the present results.

In conclusion, this study found no significant differences in the smoothness of reaching movements between real and virtual spaces. Although there are some limitations to the study, the results suggest that when reaching movements are performed from a first-person perspective using an HMD, the level of the performance can be equivalent to that in real space if physiological depth information can be provided. Although further validation of the results is required, if the same level of performance is possible in both environments, then physical rehabilitation with a high degree of freedom can be provided using virtual spaces. For example, by providing them with an environment and various situations that are closer to real life in a virtual space, patients affected by ataxic symptoms and stroke patients with motor paralysis could receive rehabilitation therapy to improve their motor skills. For example, there is a study showing the effectiveness of a finger-nose test using a tablet device as an assessment method for patients with ataxia. However, in this study, by using VR with an HMD and motion-tracking sensors, it is possible to quantitatively assess the degree of ataxia in different situations of daily life [41]. In addition, rehabilitation that also offers quantitative feedback can be conducted at home, which is expected to enhance the effectiveness of rehabilitation. Another possibility is that VR is being used to conduct basic research on motor control [42], which could also be a factor that strengthens the validity of the results. Therefore, the results of this study highlight the potential of VR technology in the field of rehabilitation and could serve as a valuable reference for future research and clinical practice. Further studies are necessary to establish the validity and effectiveness of VR-based rehabilitation approaches; however, the current findings provide a promising direction for future developments in this area. In future, it will be essential to conduct ergonomic evaluations to ensure the safety of VR implementation among patients with illnesses and disabilities.

## Figures and Tables

**Table 1:** Performance on reach movements for all targets.

	Real		VR		95%CI	T Value	Z Value	*p* Value	Effect Size
	Mean (SD)	Median [IQR]	Mean (SD)	Median [IQR]					
NJC [×10^4^]		2.73 [1.51–12.4]		2.84 [1.39–10.8]			−1.097	0.273	0.01 ^‡^
trajectory length ratio		1.13 [1.08–1.22]		1.1 [1.07–1.23]			−2.71	0.007 *	0.09 ^‡^
time of peak velocity [%]	46.6 (14.0)		46.9 (16.1)		[−3.56–2.97]	−0.179		0.858	0.02 ^†^

NJC: Normalized Jerk Cost. trajectory length ratio: Value obtained by dividing the reaching trajectory length by the shortest distance from the starting coordinates to the target coordinates. time of peak velocity: The value representing the time when the peak speed is reached, with the time at the start of the reaching operation as 0% and the time of contact with the target as 100%. * *p* < 0.05. ^†^ Cohen’s d, ^‡^ Cliff’s delta.

**Table 2:** Performance on reach movements for each target.

	Target	Real		VR		95%CI	T Value	Z Value	*p* Value	Effect Size
		Mean (SD)	Median [IQR]	Mean (SD)	Median [IQR]					
NJC [×10^4^]	① far-left		3.50 [2.30–11.1]		4.01 [2.59–34.0]			−1.183	0.237	0.10 ^‡^
	② far-middle		2.66 [1.47–14.4]		3.25 [1.67–33.3]			−0.668	0.504	0.03 ^‡^
	③ far-right		4.30 [2.29–18.1]		3.78 [1.77–14.6]			−1.82	0.069	0.11 ^‡^
	④ near-left		2.47 [1.70–13.1]		2.65 [1.71–11.2]			−0.113	0.910	0.02 ^‡^
	⑤ near-middle		1.68 [0.886–7.57]		1.51 [0.989–7.34]			−0.195	0.845	0.01 ^‡^
	⑥ near-right		2.67 [1.26–13.4]		2.36 [0.884–9.23]			−1.203	0.229	0.11 ^‡^
trajectory length ratio	① far-left		1.10 [1.06–1.18]		1.09 [1.05–1.28]			−0.072	0.943	0.08 ^‡^
	② far-middle		1.12 [1.08–1.20]		1.11 [1.08–1.28]			−0.237	0.813	0.02 ^‡^
	③ far-right		1.15 [1.11–1.22]		1.12 [1.08–1.25]			−1.656	0.098	0.14 ^‡^
	④ near-left		1.08 [1.05–1.17]		1.06 [1.04–1.14]			−2.347	0.015 *	0.24 ^‡^
	⑤ near-middle		1.13 [1.08–1.29]		1.11 [1.07–1.26]			−2.067	0.039 *	0.15 ^‡^
	⑥ near-right		1.17 [1.13–1.27]		1.15 [1.09–1.38]			−0.607	0.544	0.07 ^‡^
time of peak velocity [%]	① far-left	50.0 (11.3)		41.3 (15.3)		[1.72–15.8]	2.546		0.016 *	0.65 ^†^
	② far-middle	46.9 (12.69		43.9 (14.3)		[−4.49–10.5]	0.817		0.421	0.22 ^†^
	③ far-right		47.9 [37.0–55.2]		45.8 [38.6–54.1]			−0.524	0.6	0.07 ^‡^
	④ near-left	46.2 (11.6)		48.5 (16.4)		[−11.3–6.72]	−0.523		0.605	0.16 ^†^
	⑤ near-middle		47.4 [33.1–62.5]		49.1 [36.3–63.8]			0.524	0.6	0.05 ^‡^
	⑥ near-right	44.1 (18.9)		53.4 (15.8)			−2.132		0.042 *	0.53 ^†^

NJC: Normalized Jerk Cost. trajectory length ratio: Value obtained by dividing the reaching trajectory length by the shortest distance from the starting coordinates to the target coordinates. time of peak velocity: The value representing the time when the peak speed is reached, with the time at the start of the reaching operation as 0% and the time of contact with the target as 100%. * *p* < 0.05. ^†^ Cohen’s d, ^‡^ Cliff’s delta.

## Data Availability

The datasets analysed during this study are available from the corresponding author upon reasonable request.
